# Statement of Retraction: A novel Fas-binding outer membrane protein and lipopolysaccharide of *Leptospira interrogans* induce macrophage apoptosis through the Fas/FasL-caspase-8/-3 pathway

**DOI:** 10.1080/22221751.2023.2240210

**Published:** 2023-08-07

We, the Editors and Publishers of *Emerging Microbes and Infections*, have retracted the following article:

Peng Du, Shi-Jun Li, David M. Ojcious, Kai-Xuan Li, Wei-Lun Hu, Xu’ai Lin, Ai-Hua Sun and Jie Yan (2018) A novel Fas-binding outer membrane protein and lipopolysaccharide of *Leptospira interrogans* induce macrophage apoptosis through the Fas/FasL-caspase-8/-3 pathway, *Emerging Microbes & Infections*, 7:1, 1-17, DOI: https://doi.org/10.1038/s41426-018-0135-9.

Since publication, significant concerns have been raised about the integrity of the data in the article. An initial investigation conducted resulted in the correction of Figure 2a. However, further investigations have raised wider concerns regarding the integrity of the flow cytometry and Western blot data in the article. When approached for an explanation, the authors provided responses to our queries, but they have not sufficiently addressed our concerns. As we cannot confirm the validity of the data reported, we are therefore retracting the article.

We have been informed in our decision-making by our editorial policies and the COPE guidelines. The retracted article will remain online to maintain the scholarly record, but it will be digitally watermarked on each page as “Retracted”.

